# Failure to reach hematopoietic allogenic stem cell transplantation in patients with myelodysplastic syndromes planned for transplantation: a population-based study

**DOI:** 10.1038/s41409-022-01582-0

**Published:** 2022-02-02

**Authors:** C. Lindholm, E. Olofsson, M. Creignou, L. Nilsson, H. Gravdahl Garelius, J. Cammenga, P. Ljungman, E. Ejerblad, M. Tobiasson

**Affiliations:** 1 Center for Hematology and Regenerative Medicine, Department of Medicine Huddinge, Karolinska Institutet and Department of Hematology, Karolinska University Hospital, Stockholm, Sweden; 2 Department of Hematology, Oncology and radiation physics, Skåne University Hospital, Lund, Sweden; 3 Section of Hematology and Coagulation, Specialist Medicine, Sahlgrenska University Hospital, Göteborg, Sweden; 4 Department of Hematology, BKV and CKOC, Linköping University Hospital and Linköping University, Linköping, Sweden; 5 Department of Cellular Therapy and Allogeneic Stem Cell Transplantation, Karolinska University Hospital Huddinge and Division of Hematology, Dept of Medicine Huddinge, Karolinska Institutet, Stockholm, Sweden; 6 Department of Medical Sciences, Uppsala University, Uppsala, Sweden; Section of Hematology, Uppsala University Hospital, Uppsala, Sweden

**Keywords:** Myelodysplastic syndrome, Cancer epidemiology, Bone marrow transplantation

## Abstract

The only potential cure for patients with myelodysplastic syndrome (MDS) is allogeneic hematopoietic stem cell transplantation (HCT). However, a proportion of patients who are HCT candidates do not finally get transplanted. This population-based study aimed to characterize HCT candidates were attempting to reach HCT fail and to identify causes and risk factors for failure. Data were collected from (1) the national Swedish registry, enrolling 291 transplant candidates between 2009–2018, and (2) Karolinska University Hospital, enrolling 131 transplantation candidates between 2000 and 2018. Twenty-five % (nation-wide) and 22% (Karolinska) failed to reach HCT. Reasons for failure to reach HCT were progressive and refractory disease (47%), no donor identified (22%), identification of comorbidity (18%), and infectious complications (14%). Factors associated with failure to reach HCT were IPSS-R cytogenetic risk-group very poor, mixed MDS/MPN disease, low blast count (0–4.9%), and low hemoglobin levels (≤7.9 g/dL). Transplanted patients had a longer overall survival (OS) compared to patients who failed to reach transplantation (83 months versus 14 months; *p* < 0.001). The survival advantage was seen for the IPSS-R risk groups intermediate, high, and very high. This study demonstrated that a high proportion of HCT-candidates fail to reach HCT and underlines the difficulties associated with bridging MDS patients to HCT.

## Introduction

Myelodysplastic syndromes (MDS) and MDS myeloproliferative neoplasms (MDS/MPN) are myeloid malignancies originating in the hematopoietic stem cell compartment [1]. A third of the cases have the higher-risk disease at diagnosis, with a median survival of less than one year [2]. Allogenic hematopoietic stem cell transplantation (HCT) is the only curative regimen for these patients [3, 4].

The process of getting eligible patients who have accepted the procedure for transplantation is complex with many potential reasons which might prevent HCT. First, there might not be a suitable donor for the patient, secondly, the patient might get infections or develop co-morbidities during the pre-transplantation process, and thirdly the disease might progress to a high-proliferative disease where HCT is no longer expected to be successful. Moreover, beyond the complex medical issues encountered there are also logistical challenges e.g., referral of patients from rural hospitals to transplantation centers and obtaining the donor cells within an optimal time range. These logistical challenges must be overcome for a successful pre-transplantation process.

A proportion of patients are treated with disease-modifying treatment e.g., hypomethylating therapy such as Azacytidine (Aza) or intensive chemotherapy (ICT) during the pre-transplantation process [3, 5, 6]. The rationale of using disease-modifying treatment is to induce remission or reduce the tumor load and thereby reduce the risk of relapse [7–9]. Studies have shown that patients transplanted in remission have a better outcome after HCT compared to patients who have not achieved remission [7, 10]. However, retrospective studies have not been able to demonstrate superior outcomes for patients comparing patients pre-treated with Aza or ICT with HCT upfront, although selection bias might interfere with the results [8, 11, 12].

Data reporting the proportion of patients with an indication for HCT who finally never reach transplantation is limited. In a retrospective study from a Korean transplantation center including only patients treated with Azacytidine, 13% failed to reach HCT [7]. In another retrospective study from a single transplantation center in the US, the failure rate was 31% [13]. Two recent prospective trials have compared Aza alone vs Aza followed by HCT depending on donor availability. In the VidazaAllo trial, 33% of the patients terminated the study [14] and in the prospective study by Nakamura et al, 36% of the included patients did not get transplanted [15]. The major cause of failure in both these studies was disease progression. Data reported on failure rate is thus diverging, possibly reflecting imbalances in the patient populations. All the studies mentioned above are based on selected patient material, either from single transplantation centers or from prospective studies.

The Swedish national MDS registry includes patients with MDS and MDS/MPN nationwide from all hospitals where MDS is being diagnosed [2]. The registry has high coverage and is thus a high-quality source for population-based studies [16]. By using data from the registry, we here present the proportion of patients failing to reach HCT as well as factors predisposing for failure, in a large population-based material. We also present a detailed characterization of patients planned for HCT from a single transplantation center, providing information on the causes of failure.

## Methods

Data was collected from two cohorts of patients diagnosed with MDS or MDS/MPN with an intent to perform an HCT; (1) patients included in the National Swedish quality registry for MDS (registry data) and (2) consecutive patients from the Karolinska University Hospital (patient chart data). All included patients were classified according to the 2016 WHO classification [17]. Disease risk was stratified according to the IPSS-R scoring system [18].

### Cohort 1

The cohort consisted of all patients aged between 18 and 74, with a diagnosis of MDS or MDS/MPN reported to the National Swedish MDS registry between 2009 and 2018 [2]. The registry has a reported coverage rate of 98%, 87%, and 65% for the years 2009–2016, 2017, and 2018, respectively, at the time of data extraction November 2019. The European bone marrow transplantation (EBMT) registry and the patient charts were used to identify which patients subsequently were transplanted and which patients failed to reach transplantation. Except for this specific information, all data on disease characteristics at diagnosis was collected from the national registry. Data on missing values are reported. During the period 2009–2015, the treating physician reported if the patient was an HCT candidate or not, while from 2015 the possible options were extended with one alternative: “HCT might be performed”. Patients who were allocated to this alternative were excluded and thus only patients with a clearly documented intention to perform HCT were included. Ethical approval for studies on cohort 1 was obtained (2017/1855-31/2).

### Cohort 2

The cohort included consecutive patients aged 18–74 diagnosed with MDS or MDS/MPN at the Karolinska University hospital where the treating hematologist identified the patient as a potential transplantation candidate as documented in the patient charts. All data were collected from patient charts. Patients were diagnosed during 2000-2018, and HCT was performed between 2004 and 2019. Included patients were characterized in detail regarding disease-related parameters, pre-HCT treatment, comorbidity, marital status, and outcome. Pre-HCT treatment was categorized into Aza, ICT, or neither Aza/ICT. Comorbidity was assessed using the hematopoietic cell transplantation-comorbidity index (HCT-CI) [19]. Causes of failure to reach HCT were categorized into four categories defined as a progressive or refractory disease, no suitable donor identified, infection (uncontrolled/life-threatening infection), and identification of comorbidity (new condition or impaired functional status after the documentation of the patient being a potential transplantation candidate) making the patient unsuitable as a transplantation candidate. Ethical approval for studies on cohort 2 was obtained (2017/1090-31/4).

### Statistics

Continuous variables were expressed with the median (range). Normality was tested using the Shapiro–Wilk test. Frequency tables were used for summarizing categorical variables. Statistical methods used for association studies were the Mann–Whitney test for non-parametric distributed continuous variables and a Chi-squared test for categorical data. Binary logistic regression was used for multivariate analysis (backward conditional) of parameters associated with failure to reach HCT. Time-to-event data were analyzed using the Kaplan–Meier method. OS was defined from the time of diagnosis to the date of death or the date of the last follow-up. All statistical calculations were performed using SPSS version 25.0 (IBM, NY, United States).

## Results

### Patient population

#### Cohort 1

**Fig. 1:**
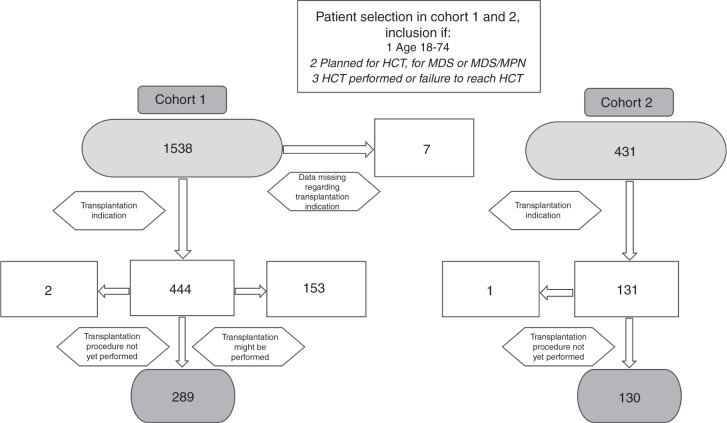
Consort diagram demonstrating the patient selection procedure. Patients were included if they were planned for HCT, were between18 and 74 years at diagnosis. Abbreviations: HCT allogeneic hematopoietic stem cell transplantation.

The Swedish national MDS registry contained a total of 3607 patients with MDS or MDS/MPN whereof 1538 were aged 18–74 years. Seven cases did not cover information regarding HCT intent and were excluded. Patients were excluded if HCT was planned but not yet performed (*n* = 2) and if allocated to the group “transplantation might be performed” (*n* = 153). In total, we included 289 patients in cohort 1 (Fig. 1). The proportion of patients aged 18–74 being envisaged for HCT, excluding patients allocated to the group “transplantation might be performed”, was 19% (*n* = 291). The median age at diagnosis was 58 years (range 18–73), and 58% were male. Fifteen % and 19% were therapy-related diseases and mixed MDS/MPN, respectively. Most patients belonged to the IPSS-R risk groups intermediate, high, or very high. Compared to cohort 2, cohort 1 had fever patients with IPSS-R intermediate and more patients with IPSS-R very high. A majority (63%) of the patients were diagnosed in a university hospital with a transplantation clinic.

#### Cohort 2

We identified 767 patients diagnosed with MDS or MDS/MPN at the Karolinska University Hospital, whereof 431 were aged between 18 and 74. The proportion of patients aged 18–74 being envisaged for HCT was 30% (*n* = 131) (Table 1 and Fig. 1). One patient was excluded since transplantation was pending but not yet performed and in total 130 patients were included in cohort 2. The median age at the decision to proceed to HCT was 60 y (range 18–74) and 63% were male. HCT was performed after a median of 175 days (range 41–171) after the documentation of the patient being an HCT candidate and 280 days (range 56–5453) after diagnosis. The median age at HCT or date when the patient was considered no longer eligible for HCT was 60 and 67 y, respectively. Most WHO 2016 classification groups were represented including 14% with therapy-related disease and 14% with mixed MDS/MPN. Most patients belonged to the IPSS-R risk groups intermediate, high, or very high. All IPSS-R karyotype risk groups were represented. The median comorbidity score (HCT-CI) [19] was 2 (range 0–10), and 65% had a registered partner. A total of 109 (84%) of the patients received disease-modifying treatment (Aza or ICT) during the pre-HCT process, while 21 (16%) patients were untreated.

**Table 1:** Patient characteristics.

	Cohort 1 (*n* = 289)	Cohort 2 (*n* = 130)	*p*-value
Age at diagnosis, median (range)	58 (18–73)	60 (18–74)	0.13
Age at HCT decision, median (range)		60 (18–74)	
Age at HCT failure, median (range)		67 (19–75)	
Age at HCT, median (range)		61 (18–75)	
Days until HCT from diagnosis, median (range)		280 (56–5453)	
Days until HCT from HCT decision, median (range)		175 (41–1171)	
Males/females, *n* (% within cohort)	168/124 (58%/42%)	82/48 (63%/37%)	0.34
Therapy related disease, *n* (% within cohort)	43 (15%)	18 (14%)	0.78
Diagnosed at University Hospital	181 (63%)		
Comorbidity index at HCT decision, median (range)		2 (0–10)	
Marital status, *n* (%)			
*Divorced*		17 (13%)	
*Married/partner*		85 (65%)	
*Single*		25 (19%)	
*Widow/widowed*		3 (2%)	
Transfusion dependent (erythrocytes), *n* (% within cohort)	143 (50%)	61 (47%)	0.61
Cellularity %, median (range)	70 (10–100)^a^	70 (20–100)	0.85
Marrow blast %	(*n* = 281)^b^		
0–4.9	103 (37%)	56 (43%)	0.21
5.0–10.0	88 (31%)	44 (34%)	0.61
10.1–19.9	90 (32%)	30 (23%)	0.06
ANC (10^9^/L)	(*n* = 285)^c^		
≥0.8	192 (67%)	95 (73%)	0.24
≤0.7	93 (33%)	35 (27%)	
Platelets (10^9^/L)	(*n* = 289)		
≥100	115 (40%)	67 (52%)	0.67
50–99	97 (33%)	33 (25%)	0.09
≤49	77 (27%)	30 (23%)	0.44
Hemoglobin (g/dL)			
≥10	133 (46%)	70 (53%)	0.14
8.0–9.9	105 (36%)	51 (39%)	0.57
≤7.9	51 (18%)	9 (7%)	0.004
WHO 2016 subgroups at diagnosis, *n* (% within cohort)	*n* = 280^d^		
Mixed MDS/MPN	52 (18.6%)	19 (14.6%)	0.33
IPSS-R risk group, *n* (% within cohort)	*n* = 265^e,f^	*n* = 126^g^	
*Very low*	5 (2%)	7 (6%)	0.05
*Low*	31 (12%)	19 (15%)	0.35
*Intermediate*	48 (18%)	42 (33%)	0.001
*High*	72 (27%)	27 (21%)	0.22
*Very high*	109 (41%)	31 (25%)	0.001
IPSS-R prognostic subgroup (karyotype), *n* (% within cohort)			
*Very good*	1 (0.5%)	2 (2%)	0.2
*Good*	104 (39%)	69 (55%)	0.004
*Intermediate*	57 (21.5%)	17 (13%)	0.06
*Poor*	39 (15%)	18 (14%)	0.91
*Very poor*	64 (24%)	20 (16%)	0.06
Treatment, all given, *n* (% within cohort)			
*Aza only*		69 (53%)	
*ICT only*		15 (12%)	
*Aza and ICT*		25 (19%)	
*Neither Aza/ICT*		21 (16%)	

Abbreviations: HCT allogeneic hematopoietic stem cell transplantation, ANC absolute neutrophil count, Plt platelet count, Hb hemoglobin level, WHO World Health Organization, MDS/MPN myelodysplastic/myeloproliferative neoplasm unclassifiable, IPSS-R revised international prognostic score system, Aza azacytidine, ICT intensive chemotherapy.

^a^Missing data in 61 patients.

^b^Missing data in 8 patients.

^c^Missing data in 4 patients.

^d^Missing data in 9 patients.

^e^Data was missing in 8 patients.

^f^16 patients could not be categorized according to IPSS/-R as they were classified as CMML-1/-2 with white blood cell count (WBC) > 12 x 109.

^g^4 patients could not be categorized according to IPSS/-R as they were classified as CMML-1/-2 with white blood cell count (WBC) > 12 x 109.

### Overlap between cohort 1 and 2

A proportion of the patients (*n* = 57) included in cohort 2 were correspondingly included in cohort 1. The remaining patients in cohort 2 (*n* = 73) were not included in cohort 1 due to (1) difference in time period (*n* = 25); (2) delay in reporting to the registry (*n* = 26); (3) HCT reported as “might be performed” in registry (*n* = 5) or reported as not planned for HCT (*n* = 17). Fourteen of the 17 patients reported as not planned for HCT were transplanted later than one year after diagnosis and most likely, these were not considered transplantation candidates upfront but later progressed and were then reevaluated. See supplementary figure S1 for details.

### Characteristics of patients who fail to reach HCT

**Fig. 2:**
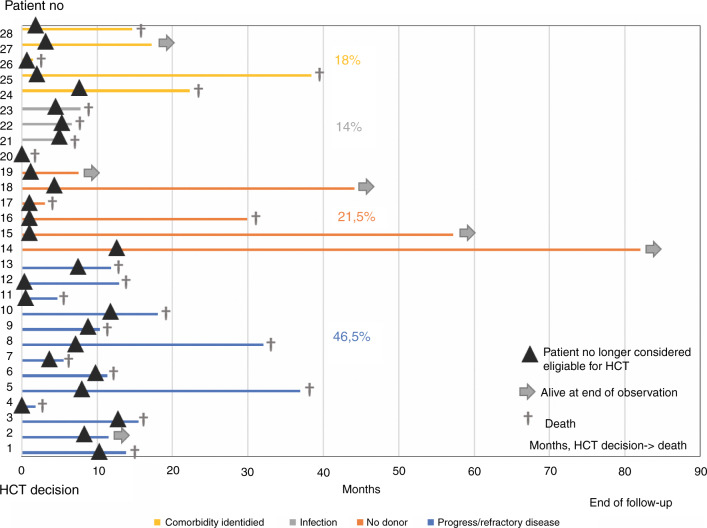
Swimmer plot illustrating patients who fail to reach HCT (*n* = 28), with the time of HCT decision as time zero. The triangle indicates the date when the patient no longer was considered eligible for HCT. The colors indicate reason for failure (yellow = identification of comorbidity, grey = infection, orange = no donor, blue = progress/refractory disease). Six patients were alive at date of last follow-up, indicated with an arrow. Abbreviations: HCT allogeneic hematopoietic stem cell transplantation.

The number of patients who failed to reach HCT was 72/289 (25%) and 28/130 (22%) in cohort 1 and 2, respectively. The time between the date when the patient was identified as a potential HCT candidate and the date when the patient was considered ineligible for HCT (cohort 2 data only) was 153 days (range 11–430 days). Cause of failure to reach HCT (cohort 2 data only) were progressive/refractory disease (*n* = 13; 47%), no suitable donor identified (*n* = 6; 21%), identification of comorbidity (*n* = 5; 18%) and infection (*n* = 4; 14%), (Fig. 2). The patients with progression/refractory disease had all received cytoreductive treatment: Aza (*n* = 6, 2–9 cycles) ICT (*n* = 2, 1-2 cycles) or Aza+ICT (*n* = 5, Aza 3–8 cycles, ICT 1–2 cycles). Median percentage of blasts in the bone marrow in these patients were 15% (range 0.5–58%) at the time of decision to cancel the HCT. Two patients who had blast counts ≤ 5 were considered no longer HCT candidates due to (a) hemophagocytic lymphohistiocytosis driven by the MDS disease, and (b) IPSS-R very poor and a remaining large *TP53* clone. No suitable donor could be identified upfront in four patients, and donor withdrawal was observed in two patients where no new donor could be identified. The cause of failure to reach HCT for the five patients with severe comorbidity was critical illness polyneuropathy (*n* = 1), severe fatigue (*n* = 2), depression (*n* = 1) and social insufficiency/alcohol-related disorder (*n* = 1). The infections resulting in failure to reach transplantation were all invasive fungal infections.

### Factors associated with failure to reach HCT

#### Univariate analysis

Factors associated with higher failure rate in cohort 1 were age (61 y in the failure group vs 57 y in the HCT group; (*p* = 0.006)), mixed MDS/MPN (36% failure rate; *p* = 0.03), IPSS-R low (39%; *p* = 0.03), lower marrow blast count (0–4.9%, (36%, *p* = 0.001)) and IPSS-R karyotype “Very Poor” (33%; *p* = 0.05). Patients with higher marrow blast count had a lower failure rate (13%, *p* = 0.003). See Table 2 for details.

**Table 2:** Univariate analysis of variables associated with failure to reach HCT.

	Cohort 1 (*n* = 289)			Cohort 2 (*n* = 130)		
Variable	No HCT (*n* = 72)	HCT (*n* = 217)	*p*-value	No HCT (*n* = 28)	HCT (*n* = 102)	*p*-value
Age at diagnosis, median (range)	61 (19–70)	57 (18–73)	0.006	65 (19–74)	59 (18–74)	0.01
Age at HCT decision, median (range)				65 (19–4)	59 (18–73)	0.02
Days until HCT decision from diagnosis, median (range)				126 (11–1681)	105 (4–5309)	0.92
Males, *n* (% of cohort)	46 (27%)	122 (63%)	0.25	19 (23%)	63 (77%)	0.55
Therapy-related disease, *n* (% of cohort)	15 (35%)	28 (65%)	0.10	6 (33%)	12 (67%)	0.19
Diagnosed at University Hospital	49 (27%)	132 (73%)	0.27			
Comorbidity index, median (range), at HCT decision				2 (0–10)	2 (0–6)	0.08
Marital status, *n* (%)						
*Divorced*				5 (29%)	12 (71%)	0.40
*Married/partner*				17 (20%)	68 (80%)	0.55
*Single*				5 (20%)	20 (80%)	0.84
*Widow/widowed*				1 (33%)	2 (67%)	0.62
*No partner*				11 (24%)	35 (76%)	0.63
Transfusion dependent (erythrocytes), *n* (% of cohort)	42 (29%)^a^	101 (69%)	0.09	14 (23%)	47 (77%)	0.71
Cellularity %, median (range)	75 (10–100)^b^	70 (10–100)^c^	0.66	70 (40–100)	70 (20–100)	0.23
Marrow blast %	*n* = 69^d^	*n* = 212^e^				
0–4.9	37 (36%)	66 (64%)	0.001	14 (25%)	24 (75%)	0.40
5.0–10.0	20 (23%)	68 (77%)	0.63	7 (16%)	37 (84%)	0.26
10.1–19.9	12 (13%)	78 (86%)	0.003	7 (23%)	23 (77%)	0.79
ANC (10^9^/L)	*n* = 72	*n* = 213^f^				
≥0.8	51 (27%)	141 (73%)	0.47	22 (23%)	73 (77%)	0.46
≤0.7	21 (23%)	72 (77%)	0.47	6 (17%)	29 (83%)	0.46
Platelets (10^9^/L)	*n* = 72	*n* = 217				
≥ 100	27 (23%)	88 (77%)	0.65	17 (25%)	50 (75%)	0.27
50–99	23 (24%)	74 (76%)	0.74	5 (15%)	28 (85%)	0.30
≤49	22 (29%)	55 (71%)	0.39	6 (20%)	24 (80%)	0.82
Hemoglobin (g/dL)	*n* = 72	*n* = 217				
≥10	24 (18%)	109 (82%)	0.01	13 (19%)	57 (81%)	0.37
8.0–9.9	31 (30%)	74 (70%)	0.17	11 (22%)	40 (78%)	0.99
≤7.9	17 (33%)	34 (67%)	0.13	4 (44%)	5 (56%)	0.08
Mixed MDS/MPN	19 (36%)^g^	33 (64%)^h^	0.03	6 (32%)	13 (68%)	0.25
Variable	Cohort 1, (*n* = 265)			Cohort 2, (*n* = 126)		
IPSS-R risk group, *n* (%)	No HCT^i,j^	HCT^k,l^	*p*-value	No HCT^m^	HCT^n^	*p*-value
*Very low*	2 (40%)	3 (60%)	0.38	0	7 (100%)	0.16
*Low*	12 (39%)	19 (61%)	0.03	4 (21%)	15 (79%)	0.97
*Intermediate*	7 (15%)	41 (85%)	0.11	9 (21%)	33 (79%)	0.99
*High*	13 (18%)	59 (82%)	0.21	5 (18%)	22 (82%)	0.68
*Very high*	28 (26%)	81 (74%)	0.46	9 (29%)	22 (71%)	0.24
Prognostic subgroup (karyotype), *n* (%)						
*Very good*	0	1 (100%)	0.58	1 (50%)	1 (50%)	0.32
*Good*	22 (21%)	82 (79%)	0.42	12 (17%)	57 (83%)	0.22
*Intermediate*	13 (23%)	44 (77%)	0.85	1 (6%)	16 (94%)	0.09
*Poor*	7 (18%)	32 (82%)	0.36	7 (39%)	11 (61%)	0.05
*Very poor*	21 (33%)	43 (67%)	0.05	6 (30%)	14 (70%)	0.31
All given treatment				No HCT	HCT	*p*-value
*Aza only*				15 (22%)	54 (78%)	0.95
*ICT only*				4 (27%)	11 (73%)	0.60
*Aza and ICT*				7 (28%)	18 (72%)	0.38
*Neither Aza/ICT*				2 (10%)	19 (90%)	0.14

Abbreviations: HCT allogeneic hematopoietic stem cell transplantation, ANC absolute neutrophil count, Plt platelet count, Hb hemoglobin level, WHO World Health Organization, IPSS-R revised international prognostic score system, Aza azacytidine, ICT intensive chemotherapy.

^a^Data missing in 1 patient.

^b^Data missing in 18 patients.

^c^Data missing in 43 patients.

^d^Data missing in 3 patients.

^e^Data missing in 5 patients.

^f^Data missing in 4 patients.

^g^Data missing in 2 patients.

^h^Data missing in 7 patients.

^i^Data missing in 3 patients.

^j^7 patients could not be categorized according to IPSS/-R as they were classified as CMML-1/-2 with white blood cell count (WBC) > 12 x 109.

^k^Data missing in 5 patients.

^l^9 patients could not be categorized according to IPSS/-R as they were classified as CMML-1/-2 with white blood cell count (WBC) > 12 x 109.

^m^1 patient could not be categorized according to IPSS/-R as they were classified as CMML-1/-2 with white blood cell count (WBC) > 12 x 109.

^n^3 patients could not be categorized according to IPSS/-R as they were classified as CMML-1/-2 with white blood cell count (WBC) > 12 x 109.

In cohort 2, factors associated with higher failure rate were age (65 vs 59 y; *p* = 0.01), IPSS-R karyotype “Poor” (39%; *p* = 0.05). The other factors associated with failure rate in cohort 1 could not be replicated in cohort 2. Data on pre-HCT treatment was only available for cohort 2. There was no significant difference in failure rate between patients treated with Aza or patients treated with ICT (22%, *p* = 0.95 and 28%, *p* = 0.27). Twenty-one patients were planned for HCT upfront without any disease-modifying treatment prior to HCT. Two of these did not get transplanted due to no identified donor.

#### Multivariate analysis

Multivariate analysis of failure rate was performed in both cohorts separately and included all variables with *p*-value ≤ 0.1 in the univariate analysis. In cohort 1, the included variables were: age at diagnosis, marrow blast category 0–4.9% and > 0%, hemoglobin level category ≥10 g/dL, WHO 2016 subgroup mixed MDS/MPN, therapy-related disease, and IPSS-R karyotype very-poor. Blast count 0–4.9%, very poor cytogenetics and age remained independently associated with failure to reach HCT (OR 2.59, *p* = 0.003, OR 2.0, *p* = 0.05 and OR 1.05, *p* = 0.003, respectively) while hemoglobin ≥10 g/dL at diagnosis were independently associated with a lower risk of failure (OR 0.45, *p* = 0.02). See Table 3 for details.

**Table 3:** Multivariate analysis of variables associated with failure to reach HCT.

	Cohort 1 (*n* = 289)			Cohort 2 (*n* = 130)		
Variable	OR	95% CI	*p*-value	OR	95% CI	*p*-value
Age at diagnosis	1.05	1.02–1.09	0.003	1.05	1.00–1.09	0.04
Therapy-related disease	1.74	0.79–3.87	0.17			
Comorbidity index				1.2	0.98–1.53	0.07
Marrow blast 0–4.9%	2.59	1.39–4.82	0.003			
Marrow blast 10.1–19.9%	0.50	0.21–1.19	0.12			
Hemoglobin ≥ 10 g/dL	0.45	0.24–0.87	0.02			
Hemoglobin ≤ 7.9 g/dL				2.9	0.62–12.8	0.18
WHO2016 Mixed MDS/MPN	1.4	0.56–3.39	0.48			
IPSS-R prognostic subgroup (karyotype) intermediate				0.35	0.04–2.95	0.33
IPSS-R prognostic subgroup (karyotype) poor				2.44	0.80–7.43	0.12
IPSS-R prognostic subgroup (karyotype) very poor	2.0	1.01–4.12	0.05			

Abbreviations: WHO World Health Organization, IPSS-R revised international prognostic score system.

The included variables in cohort 2 were: age at diagnosis, comorbidity index, Hb ≤ 7.9, IPSS-R karyotype intermediate, and poor. Only age was independently associated with a higher risk of failure (OR 1.05, *p* = 0.04).

### Survival

#### Cohort 1

**Fig. 3:**
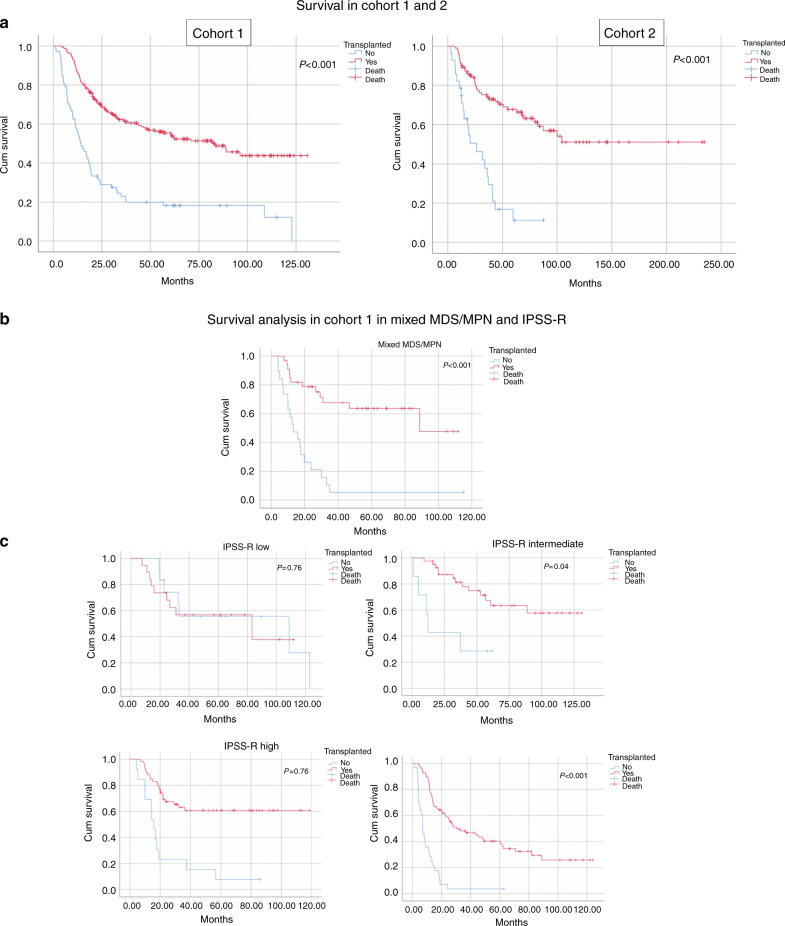
Panel a demonstrates OS in cohort 1 and 2. Panel **b** demonstrates OS in cohort 2 with regards to WHO 2016 classification. Panel **c** demonstrates OS in cohort 2 with regards to IPSS-R. Abbreviations: Cum survival Cumulative survival, WHO World Health Organization, IPSS-R revised international prognostic score system, MDS/MPN myelodysplastic/myeloproliferative neoplasm.

After a median follow up from diagnosis of 29 (range 1–131) months, 129 patients were still alive. Estimated median OS was 44 months, with a longer median OS for transplanted patients vs patients who failed to reach HCT (83 vs 14 months; *p* < 0.001). There was a survival benefit for transplanted patients in all IPSS-R risk groups except for IPSS-R very low and low where no significant difference was observed (Fig. 3). Moreover, there was a survival benefit for transplanted patients over failure patients also in the MDS/MPN risk group.

#### Cohort 2

After a median follow-up from diagnosis of 41.5 (range 3–235) months after HCT decision, 70 patients were still alive. Estimated median OS was 82 months, with a longer median OS for transplanted patients (105 vs 26 months; *p* < 0.001). Twenty-seven patients (25%) relapsed after HCT after a median of 10 (range 3–56) months. Eighteen patients died due to non-relapse mortality after a median of four (range 2–51) months.

Six patients who failed to reach HCT were alive at end of observation. The cause of failure in these patients were lack of donor (*n* = 4), progressive disease (*n* = 1) and identification of comorbidity (*n* = 1). The patient with progression had an observation time of three months after the decision to cancel the transplantation. The patient with comorbidity had developed severe depression. Twenty-two patients who failed to reach HCT were dead at the last follow-up, with a median time between the date when the patient was no longer considered a candidate for HCT and death of 1.4 months (0–36 months). Cause of death was disease progression (*n* = 14, 64%), infection (*n* = 7, 32%), and pulmonary embolism (*n* = 1, 4%), see Fig. 2.

## Discussion

The transplantation process for patients with MDS is a challenging task and includes both patient and donor-related issues: the disease needs to be under control, a donor needs to be identified and the patient should not acquire severe infections or co-morbidities during the pre-transplantation process. In addition, several logistical challenges must be overcome. The outcome for patients eligible for HCT is often presented as survival after HCT, but we know that a proportion of patients do not reach transplantation. Previous studies on the proportion of patients not being transplanted mainly consist of selected cohorts from single transplantation centers or prospective studies [7, 13–15]. This is to our knowledge the first study analyzing outcomes for patients planned for HCT in a population-based material. We report a large proportion of patients not reaching HCT which underlines the difficulties to take patients through the pre-transplantation process and the importance of careful surveillance during this process.

A limitation of this study is the inability to identify patients in cohort 1 who are recorded as non-HCT candidates in the registry due to low-risk disease, but then experience disease progression and are reconsidered as HCT candidates. Another obvious limitation of the data in cohort 1, is recall bias when doctors are reporting to the registry, where patients who might already have been transplanted upon registration are reported as “HCT planned” while failure patients might be recorded as “HCT not planned” despite an initial intent to transplant these patients. Since cohort 2 includes detailed data from patient charts, the limitations of cohort 1 described above do not exist for cohort 2.

The factors associated with failure to reach HCT can be divided into patient-related (e.g., age), disease-related (e.g., risk profile, hemoglobin level), donor-related (no donor identified or donor withdrawal). Potentially there are also physician-related factors and center bias factors. The intriguing finding that patients with blast count < 5% and patients with the lower-risk disease have a higher failure rate in cohort 1 might be attributed to a more passive pre-transplantation work-up and delayed HCT or due to less careful disease surveillance. This is obviously speculation, and other reasons for failure to reach HCT e.g., revised transplantation indication for patients admitted to transplantation centers from rural hospitals, revised transplantation indication due to suboptimal donor or patient refusal, might be important. Interestingly, there was no significant difference in the failure rate for patients diagnosed in a rural hospital compared to patients diagnosed at a university hospital (27%; vs 21%; *p* = 0.27) which would argue for a similar selection of transplantation candidates at Swedish rural hospitals compared to university hospitals.

The most frequent cause of failure to reach HCT in this cohort is progressive/refractory disease which is in line with the two recently published prospective studies [14, 15]. Our study underscores the importance of active treatment and disease surveillance for this group of patients. Potentially, newer treatments can reduce the number of patients who fail to reach HCT due to progressive/refractory disease.

A small number of patients failed to reach HCT due to a lack of donor. Due to the recent development of using haploidentical-donors, the number of patients not being possible to transplant because of lack of donor, could probably be decreased [20].

We observed an OS benefit for transplanted patients in both cohorts. The difference in survival might partly be explained by patient selection, where patients who fail to reach HCT might be more fragile and/or have a more serious disease. Interestingly, the survival difference for patients with IPSS-R intermediate, where HCT is controversial, demonstrates a large survival benefit for patients being transplanted. Again, selection bias could contribute but the large difference between the groups might support HCT for these patients. Conversely, there is no survival benefit for patients with IPSS-R low and very low and a careful selection of patients who are candidates for HCT should be applied for these patients [21].

In summary, we have in a population-based cohort shown that there is a significant proportion of patients failing to reach HCT underlining the difficulties to bring patients to HCT. New tools such as new treatments, new donor sources, and new surveillance methods can hopefully reduce the rate of patients failing to reach HCT.

## Supplementary information

Supplemental Material
