# Supplementary material for: Failure to reach hematopoietic allogenic stem cell transplantation in patients with myelodysplastic syndromes planned for transplantation: a population-based study
Source: Bone Marrow Transplant. 2022 Feb 2;57(4):598–606. doi: 10.1038/s41409-022-01582-0 (PMC8993688; doi:10.1038/s41409-022-01582-0)

**Supplementary data**

**Figure legends**

**Supplementary figure 1;** Consort diagram demonstrating patients in cohort 2 (n=73) who were not included in cohort 1. Reasons for no inclusion in the population-based cohort were: 1) difference in time period (n=25); 2) delay in reporting to the registry (n=26); 3) HCT reported as “might be performed” in registry (n=5) or reported as not planned for HCT (n=17). Abbreviations: HCT; allogeneic. hematopoietic stem cell transplantation.

**Figures**

**Supplementary figure 1**


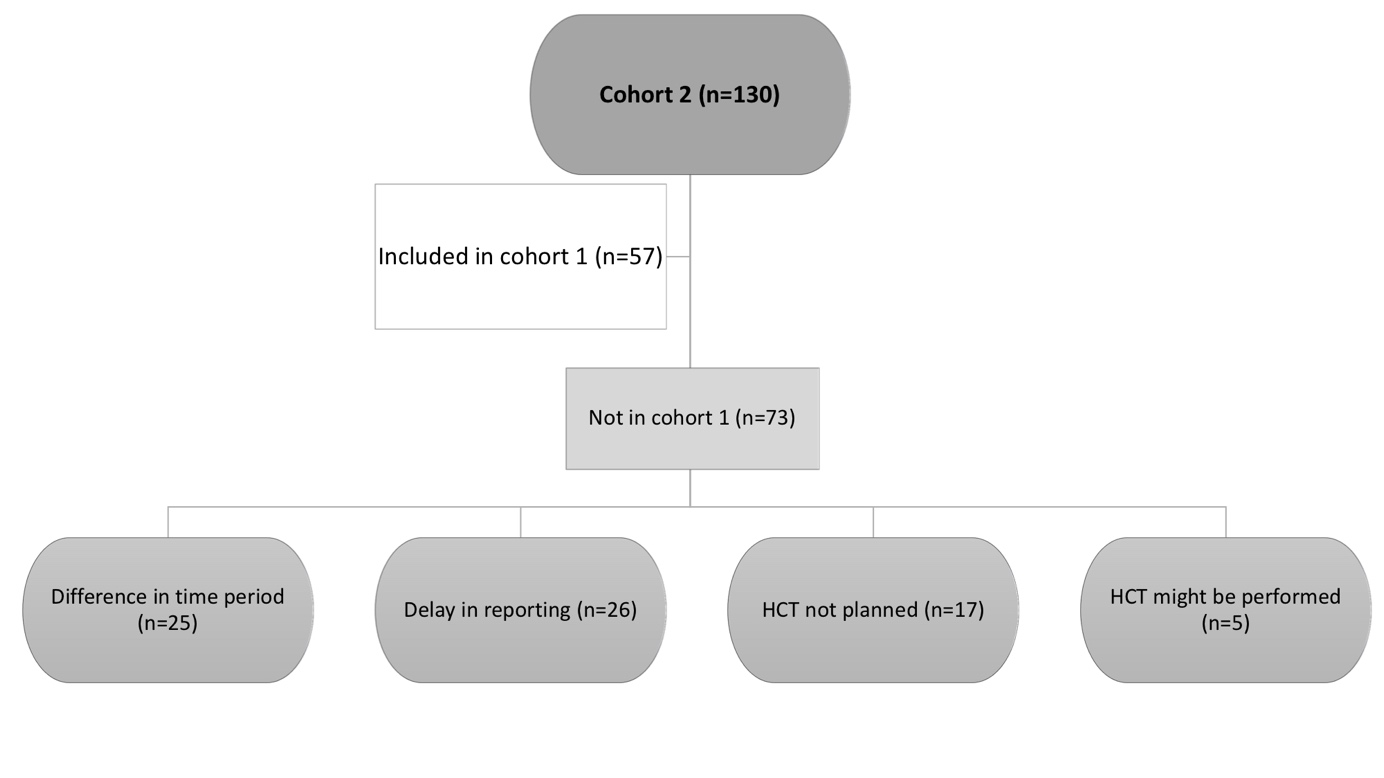

Supplement: Supplementary file 1 — Supplemental Material [file 41409_2022_1582_MOESM1_ESM.docx]
